# Factors associated with restrained eating and validation of the Arabic version of the restrained eating scale among an adult representative sample of the Lebanese population: a cross-sectional study

**DOI:** 10.1186/s40337-019-0254-2

**Published:** 2019-07-17

**Authors:** Sylvia Saade, Souheil Hallit, Chadia Haddad, Rabih Hallit, Marwan Akel, Karl Honein, Maria Akiki, Nelly Kheir, Sahar Obeid

**Affiliations:** 1 School of Pharmacy, Lebanese International University, Beirut, Lebanon; 2 Faculty of Medicine and Medical Sciences, Holy Spirit University of Kaslik (USEK), Jounieh, Lebanon; 3 INSPECT-LB: Institut National de Santé Publique, Epidemiologie Clinique et Toxicologie, Beirut, Lebanon; 4 Psychiatric Hospital of the Cross, 60096, Jal Eddib, Lebanon; 5 Faculty of Pedagogy, Université de la Sainte Famille, Batroun, Lebanon; 6 Faculty of Philosophy and Human Sciences, Holy Spirit University of Kaslik (USEK), Jounieh, Lebanon; 7 Faculty of Pedagogy, Lebanese University, Beirut, Lebanon

**Keywords:** Restrained eating, Body dissatisfaction, Attachment, Emotional eating

## Abstract

**Background:**

Previous research suggests that restrained eating is problematic in Lebanon and is associated with the occurrence of clinically diagnosed eating disorders. Because of the alarming prevalence and severity of these disorders, the aim of this study is to investigate factors that may contribute to restrained eating in adults among a representative sample of the Lebanese population.

**Methods:**

This is a cross-sectional study conducted between January and May 2018; 811 adult participants were enrolled from all Lebanese districts. The Dutch Restrained Eating scale was used to measure body disturbance. The factors that were assessed in the questionnaire were body dissatisfaction, self-esteem, perceived stress, anxiety, depression, emotion regulation, emotional eating and adult attachment styles.

**Results:**

The mean age of the participants was 27.59 ± 11.76 years, and included 66.5% females. In the absence of a cutoff value for the Dutch Restrained Eating scale, we took the median (2.6) as the cutoff value. The results showed that 391 (48.3%) had restrained eating. The Dutch Restrained Eating scale items converged over a solution of one factor that had an Eigenvalue over 1, explaining a total of 60.69% of the variance (Cronbach’s alpha was high =0.928). The linear regression results, taking the Dutch restrained eating scale as the dependent variable, showed that being a female (Beta = 0.31), increased age (Beta = 0.01), higher body mass index (Beta = 0.01), an intermediate monthly income (Beta = 0.25), higher body dissatisfaction scores (Beta = 0.03), higher adult anxiety attachment style (Beta = 0.008), higher emotion regulation cognitive reappraisal facet (Beta = 0.01), feeling pressure from TV/magazine to lose weight (Beta = 0.45) and practicing sport activities (Beta = 0.41) were associated with higher restrained eating scores.

**Conclusion:**

Our findings show that numerous factors are associated with restrained eating in the Lebanese community. They include body dissatisfaction, cognitive reappraisal, female gender, eating attitudes, social media pressure and adult attachment. Therefore, the development of prevention strategies directed at an improved body image perception and increasing knowledge about factors that might influence this body image and critical thinking regarding media images is warranted, with the ultimate goal of promoting healthier choices in the Lebanese population.

## Introduction

Eating disorders are classified as the third most prevalent chronic disease among young females in the United States [1]. In Lebanon, a study published in 2017 showed that bulimia nervosa (46.1%), followed by anorexia nervosa (39.4%) and binge eating (14.4%) were the most common eating disorders [2], with these numbers expected to grow each year.

The restrained eating theory was first introduced by Herman and Mack who described the predisposition of individuals to limit food consumption to reach weight loss [3]. However, several studies have suggested that chronic or excessive restriction may have a rebound effect and actually cause weight gain [4, 5] due to the disinhibition of eating behaviors in the presence of life stressors compared to when not distressed [6]. In fact, certain events such as stress [7] or pleasurable foods [8] may disrupt the individual’s intent to diet. Consequently, repetitive successions of restrained eating and disinhibition may lead to a vulnerable weight cycle [9]. In Lebanon, one study showed that 30% of university students tried to lose weight although 71% did not have weight excess [10], whereas a second one showed that 52.9% of young adults wanted to lose weight, despite only 6.1% of them being overweight [11]. This previously conducted research suggests that restrained eating is problematic in Lebanon and is worthy of further investigation into the factors that lead to restrained eating in this population. Below is a discussion of past research on potential factors that have shown to predict restrained eating in other populations, particularly in Western societies in the United States and Europe. Several socio-demographic factors are implicated in the development and maintenance of eating disorders. Being a female, earning an intermediate to high income, having a low education level and increased physical activity have been associated with higher restrained eating behaviors [12]. Moreover, although research on eating disorders has focused on children and young adolescents, recent studies have demonstrated that eating disorders are becoming increasingly common at midlife or beyond [13]. It is well established that BMI influences eating and weight directed patterns. Past research has demonstrated that higher BMI is associated with increased restraint and emotional eating [14, 15], including a study conducted in Lebanon [16]. Furthermore, studies have revealed the significance of interpersonal relationships as a development and maintaining feature in eating disorders [17, 18]. Kiriike et al. conducted a study in 2014 suggesting a positive link between married women and disordered eating, due to the major role of marital discord [19]. Virtually all eating disorders concepts refer to body image, defined as “self-appraisals and emotional experiences about one’s physical appearance” [20]. Body dissatisfaction, defined as “negative feelings about the body”, has significant repercussions on one’s psychological functioning and quality of life and characterizes individuals with eating disorders [20]. It appears to be associated with one’s attempts to restrain eating [21]. Body shape concerns are even more prevalent than eating disorders among females than males [22]. Although the intention to control the weight is present, restrained eaters often fail in pursuing their diet and frequently consume larger quantities of high-fat foods that they normally forbid themselves from eating [23]. Research revealed that the individual’s satisfaction with his/her body image fluctuates, especially in people with concerns about their weight and shape [24].

Parents, family members and friends may pressure a person to seek a thin ideal body [25]. Same applies for the media, including but not limited to newspapers, magazines, TV, Internet, etc. [26], with previous research supporting an association between media pressure and the development and treatment outcomes of eating disorders [27]. The attachment theory is recognized for its high value in multiple areas of psychological functioning. Its concept is commonly used in emotional, social, and interpersonal problems and was associated with the development and maintenance of eating disorders and psychological disorders, such as anxiety, self-esteem, and depression [28]. On another hand, people instinctively use emotion regulation strategies to deal with stressful situations multiple times during each day. Individuals capable of regulating their feelings in a flexible way have the abilities to experience, distinguish, reduce and control these affective states [29]. It has been suggested that individuals resort to inappropriate eating behaviors as an escape route and a way to reduce negative emotions, due to a lack of effective coping strategies [30]. Self-esteem was also linked to restrained eating and was found to be a mediating factor between body dissatisfaction and restrained eating [31]. Many psychological indicators have been linked to eating pathology, including stress, anxiety and depression [32, 33]. Emotion is also considered as an important psychological factor that can be differentially induced by or related to stress and consequently can affect the eating behavior outcomes [34]. One hypothesis to explain the correlation between stress and restrained eating is that stress is a cognitive demanding task that requires a big mental capacity from the restrained eater to the point that he/she no longer thinks about restricting their eating [35]. In a previous study conducted in Lebanon, the relationship between body dissatisfaction and restrained eating was significantly affected by anxiety but not by depression or stress [36]. In view of all what has been said and in view of the lack of data concerning restrained eating in Lebanon, we decided to conduct this study in order to linguistically validate the Dutch Restrained Eating scale in Arabic and evaluate the factors shaping restrained eating among an adult representative sample of the Lebanese population.

## Methods

### Participants

This cross-sectional study, conducted between January and May 2018, which enrolled 811 community dwelling adult participants using a proportionate random sample from all Lebanese governorates (Beirut, Mount Lebanon, North, South and Bekaa). Each governorate is divided into Caza (stratum); two villages were randomly selected from the list of villages provided by the Central Agency of Statistics in Lebanon. Participants were randomly selected from each village. In each selected village the questionnaire was distributed randomly to the households, based on random sampling technique to select the included house. Those who accepted to participate in the study were invited to fill out the questionnaire.

### Minimal sample size calculation

The sample size was calculated using Epi-Info by considering a population size of 6.000.000 Lebanese, an assumed prevalence of 50% of restrained eating among the general population in the absence of similar studies in the country and a 95% confidence level. The minimal sample size needed was 384.

### Questionnaire

The interview was done in the participant’s house and in Arabic, the native language of Lebanon. The first part assessed the sociodemographic details of the participants (age, gender, marital status, educational level, monthly income (divided into four levels: no income, low income < 1000 USD; intermediate income 1000–2000 USD; and high income > 2000 USD)). The body mass index (BMI) was calculated by dividing the person’s self-reported weight (in Kg) by the self-reported height in meters squared (m^2^). Alcohol, tobacco and caffeine intake as well a family history of eating disorders were categorized into dichotomous variables (yes/no). Also, questions about “receiving comments from the family concerning losing weight”, “being insulted”, “being physically abused”, and “being sexually abused” were categorized into dichotomous variables (yes/no). Information about the use of illegal drug substances use/addiction were self-reported by each participant. The physical activity index, based on responses to a series of questions about the intensity, frequency and duration of participation in leisure-time physical activity, is a frequently used indicator of physical activity at the population level. The Total Physical Activity Index was calculated by multiplying the intensity, duration and frequency of daily activity [37].

The final part included the scales used in this study as follows:

### Dutch restrained eating scale

The Dutch Restrained Eating Scale is a ten-item scale that assesses the frequency of dieting behaviors by using a five-point Likert scale, ranging from 1 (never) to 5 (always). Examples given of the asked questions: “When you have put on weight, do you eat less than you usually do?”, “Do you watch exactly what you eat?”, “Do you deliberately eat less in order not to become heavier?”, “Do you take into account your weight with what you eat?”. The score for this scale was obtained by dividing the total items score by the total number of items. A higher score would indicate a higher degree of restrained eating [38]. In this study, the Cronbach’s alpha was 0.928.

### Body dissatisfaction subscale of the eating disorder inventory-second version (EDI-2)

The EDI-2 is a 91 item self-report instrument that is used to assess psychological characteristics relevant to eating disorders [39]. It consists of 11 subscales: (1) drive for thinness, (2) bulimia, (3) body dissatisfaction, (4) ineffectiveness, (5) perfectionism, (6) interpersonal distress, (7) introspective awareness, (8) maturity fears, (9) asceticism, (10) impulse regulation, (11) and social insecurity [39]. Examples given of the asked questions: “I find my thighs too big”, “I find my hips too wide ”, “I find my hips just on the right size”, “I find my thighs just on the right size”. In the present study, the body dissatisfaction score was measured from the eating disorder inventory (EDI-2) subscale. The scale assesses the levels of dissatisfaction with the overall body shape and specific body parts. The body dissatisfaction subscale consists of nine items, measured in 4-point Likert scales, ranging from 0 (sometimes, rarely, never) to 3 (always). Five questions were reversed while doing the score calculation. The total score was calculated by summing the nine items. The total score ranged from 0 to 27. Higher scores are indicative of greater body dissatisfaction [39]. In this study, the Cronbach’s alpha was 0.779.

### The Rosenberg self-esteem scale

The Rosenberg self-Esteem scale is composed of ten items and is used to assess beliefs and attitudes regarding general self-worth. The answers were graded using a four point Likert scale, with answers ranging from 1 (strongly disagree) to 4 (strongly agree). Examples given of the asked questions: “On the whole, I am satisfied with myself”, “I feel I do not have much to be proud of”, “I certainly feel useless at times”, “I wish I could have more respect for myself”. Five questions (3, 5, 8, 9, and 10) were reversed while doing the score calculation. The total score is calculated by summing the 10 items. The scale ranged from 10 to 40. Higher scores would indicate higher self-esteem [40]. In this study, the Cronbach’s alpha was 0.759.

### Perceived stress scale (PSS)

The PSS is a ten-item scale widely used for measuring the perception of stress during the last month [41]. The questions answers ranged from never (0) to almost always (4). Items 4, 5, 7, and 8 are reversed items. Examples given of the asked questions: “In the last month, how often have you been upset because of something that happened unexpectedly?”, “In the last month, how often have you felt confident about your ability to handle your personal problems?”, “In the last month, how often have you found that you could not cope with all the things that you had to do?”, “In the last month, how often have you felt difficulties were piling up so high that you could not overcome them?”. The total score was calculated by summing the 10 items, with higher scores indicating more perceived stress [41]. The total score ranged from 0 to 40. In this study, the Cronbach’s alpha was 0.709.

### Hamilton anxiety rating scale (HAM-A)

The HAM-A is one of the first rating scales to measure the severity of perceived anxiety symptoms. It consists of fourteen symptom-defined elements, identifying both psychological and somatic symptoms. Each item is scored on a basic numeric scoring of 0 (not present) to 4 (severe). Examples given of the items: “Anxious mood”, “Insomnia”, “depressed mood”, “fears”. The total score, calculated by summing the 14 items, ranged from 0 to 56, with higher scores indicating higher anxiety [42].The validated version of this scale was used in this study [reference: Souheil Hallit, et al., Clinical Epidemiology and Global Health, 10.1016/j.cegh.2019.02.002]. In this study, the Cronbach alpha’s was 0.912.

### Hamilton depression rating scale (HAM-D)

The HAM-D, validated in Arabic [43], was used to assess the severity of depression in patients who are already diagnosed as depressed. Examples given of the items used: “depressed mood”, “feelings of guilt”, “suicide”, “insomnia initial”. The total score is based on the sum of the first 17 items. Higher scores would indicate higher depression [44]. In this study, the Cronbach’s alpha was 0.879.

### Emotion regulation Questionnaire (ERQ)

The ERQ, a ten-item scale, is used to measure respondents’ tendency to regulate their emotions in two ways: (1) Cognitive Reappraisal and (2) Expressive Suppression. Answers scores ranged from 1 (strongly disagree) to 7 (strongly agree). Examples given of the asked questions: “When I want to feel more positive emotion (such as joy or amusement), I change what I’m thinking about”, “When I’m faced with a stressful situation, I make myself think about it in a way that helps me stay calm”, “When I want to feel more positive emotion, I change the way I’m thinking about the situation”, “When I am feeling negative emotions, I make sure not to express them”. The sum of items 1, 3, 5, 7, 8, 10 make up the Cognitive Reappraisal facet and the sum of items 2, 4, 6, 9 make up the Expressive Suppression facet. Each facet’s scoring is kept separate. The higher the scores, the greater the use of the emotion regulation strategy [45]. In this study, the Cronbach’s alpha values for the Cognitive Reappraisal facet and for the Expressive Suppression facet were 0.744 and 0.732 respectively.

### Emotional eating scale (EES)

The EES scale is composed of twenty five items, with three derived subscales: anger, anxiety and depression. Participants rate the extent to which certain feelings lead to the urge to eat, using a five point Likert scale ranging from 0 (no desire to eat) to 4 (an overwhelming urge to eat). Examples given of the asked items: “Resentful”, “discouraged”, “rebellious”, “worried”. The total score is calculated by summing the answers of all items. The highest possible score is 100. Higher scores indicate a reliance on using food to help managing emotions [46]. In this study, the Cronbach’s alpha was 0.957.

### State adult attachment measure (SAAM)

The SAAM measures three different aspects of the adult attachment: security, anxiety and avoidance. It consists of twenty one Likert scale questions ranging from 1 (strongly disagree) to 7 (strongly agree). Examples given of the asked questions: “I feel like I have someone to rely on”, “I want to talk with someone who cares for me about things that are worrying me”, “I really need someone’s emotional support”, “I have mixed feelings about being close to other people”. The total score is calculated by summing the 21 items. The highest possible score is 147 indicating a high features of attachment [47]. In this study, the Cronbach’s alpha was 0.827.

### Forward and back translation procedure

All scales underwent a forward translation, which was first conducted by a single bilingual translator, whose native language is Arabic and is fluent in English. An expert committee formed by healthcare professionals and a language professional verified the Arabic translated version. A backward translation was then performed by a native English speaker translator, fluent in Arabic and unfamiliar with the concepts of the scales. The back-translated English questionnaire was subsequently compared to the original English one, by the expert committee, aiming to discern discrepancies and to solve any inconsistencies between the two versions. The process of forward-back translation was repeated until all ambiguities disappeared.

### Test-retest reliability of the Dutch restrained eating scale

To assess test-retest reliability of the scale, 100 individuals of the first sample answered the questionnaire twice. The time between test and re-test reproducibility examination averaged approximately 10 days.

### Statistical analysis

The SPSS software version 23 was used to conduct data analysis. A Cronbach’s alpha was recorded for reliability analysis for all the scales. Intraclass correlation coefficient (ICC) were also used for reliability analysis of the test-retest. A good reproducibility was noted when ICC > 0.7 (Terwee et al., 2007). A descriptive analysis were done using the absolute frequency and percentages for categorical variables and mean and standard deviation for quantitative measures. Two different methods were used to confirm the Dutch Restrained Eating scale construct validity. First, a factor analysis was run using the principal component analysis technique on half the sample (*N* = 406). Since the extracted factors were found to be significantly correlated, the promax rotation technique was used. To ensure the model’s adequacy, the Kaiser-Meyer-Olkin measure of sampling adequacy and Bartlett’s test of sphericity were calculated. Factors with an Eigen value higher than one were retained. Moreover, Cronbach’s alpha was recorded for reliability analysis for each scale. Second, a confirmatory factor analysis was carried out on the second half of the original sample (*N* = 405). To assess the structure of the instrument the maximum likelihood method for discrepancy function was used. Several goodness-of-fit indicators were reported: the Relative chi square (× 2/df), the Root Mean Square Error of Approximation (RMSEA), the Goodness of Fit Index (GFI) and the Adjusted Goodness of Fit Index (AGFI). The index of goodness of fit was calculated by the value of × 2 divided by the degrees of freedom (× 2/df) (cut-off values < 2–5). The RMSEA tests the fit of the model to the covariance matrix. As a guideline, values of< 0.05 indicate a close fit and values below 0.11 an acceptable fit. The GFI and AGFI are chi-square-based calculations independent of degrees of freedom. The recommended thresholds for acceptable values are ≥0.90 [48]. To assess the associations with the continuous restrained eating score, Pearson correlation analyses were used with the continuous variables, and the Student t-test and ANOVA F tests were used for categorical variables with two or more levels, respectively. Since the correlation coefficients were all found to be< 0.8, these variables were judged to be interrelated but not multi-colinear, and we were able to use them together in regression models. Three hierarchical stepwise linear regressions were conducted, taking the Dutch Restrained Eating Scale as the dependent variable. All variables that showed a *p* < 0.1 in the bivariate analysis were considered as important variables to be entered in the model in order to eliminate the potential confounding factors as much as possible [49]. These three models were built by adding variables to the previous model at each step in order to determine that the newly added variables would improve the proportion of explained variance of the dependent variable by the model (improve in adjusted R^2^). The stepwise method was used to simultaneously remove variables that were weakly correlated to the dependent variable. Thus, the final variables kept in the model explain better the distribution. A *p*-value less than 0.05 was considered significant.

## Results

### Sociodemographic characteristics

Out of 1000 questionnaires distributed, 806 (80.6%) were completed and collected. The sociodemographic characteristics of the participants are summarized in Table 1. The results showed that the mean age of the participants was 27.59 ± 11.76 years, and included 66.5% females. The majority (73.2%) had a university level of education, were single (67.0%), with a low monthly income (77.9%). Almost all participants drank caffeine (90%), 30.8% were smokers and 4.2% were alcohol users. The majority practiced physical activities (62.4%). The mean BMI of the participants was 18.09 ± 11.68. In the absence of a cutoff value for the Dutch Restrained Eating scale, we took the median (2.6) as the cutoff value. The results showed that 391 (48.3%) had restrained eating.

**Table 1:** Sociodemographic characteristics of the study sample

	Frequency (%)
Gender	
Male	270 (33.5%)
Female	536 (66.5%)
Marital status	
Single	581 (71.6%)
Married	230 (28.4%)
Education level^a^	
Primary	24 (3.1%)
Complementary	61 (7.8%)
Secondary	125 (15.9%)
University	574 (73.2%)
Monthly income	
No income	340 (45.1%)
< 1000$	247 (32.8%)
1000 – 2000 $	117 (15.5%)
> 2000 $	50 (6.6%)
Tobacco use	
Yes	246 (30.8%)
No	554 (69.2%)
Drug addiction	
Yes	7 (0.9%)
No	751 (99.1%)
Alcohol users	
Yes	32 (4.2%)
No	724 (95.8%)
Caffeine	
Yes	721 (90.0%)
No	80 (10.0%)
Practicing sport activities	
Yes	490 (62.4%)
No	295 (37.6%)
	Mean ± SD
Age (in years)	27.59 ± 11.76
Body Mass Index (Kg/m^2^)	18.09 ± 11.68

^a^Primary level of education refers to less than 6 years of study; complementary level refers to more than 6 years of study but less than 10 years

Some numbers do not add up to 811 (the total number of the recruited participants) because of missing values in those variables

### Factor analysis

The factor analysis for the Dutch Restrained Eating scale was run over half of the sample (subsample 1; *n* = 406). All items could be extracted from the list, none of the items was removed since no items over-correlated to each other (r > 0.9), had a low loading on factors (< 0.3) or because of a low communality (< 0.3). The Dutch Restrained Eating scale items converged over a solution of one factor that had an Eigenvalue over 1, explaining a total of 60.69% of the variance. A Kaiser-Meyer-Olkin measure of sampling adequacy of 0.938 was found, with a significant Bartlett’s test of sphericity (*p* < 0.001). According to the promax rotated matrix, the components are summarized in Table 2. Moreover, a high Cronbach’s alpha was found for the full test (0.928). The exploratory factor analysis conducted on the subsample 2 (*N* = 405) showed similar results in terms of the number of factors (1 factor) and similar Cronbach’s alpha value (α = 0.921).

**Table 2:** Promax rotated matrix of Dutch Restrained Eating scale conducted on subsample 1 (*N* = 403)

	Items	Factor 1
Do you deliberately eat less in order not to become heavier?	7	0.822
How often do you try not to eat between meals because you are watching your weight?	8	0.814
How often in the evenings do you try not to eat because you are watching your weight?	9	0.811
When you have eaten too much, do you eat less than usual the following day?	6	0.804
Do you try to eat less at meal times than you would like to eat?	2	0.787
Do you take into account your weight with what you eat?	10	0.785
How often do you refuse food or drink offered because you are concerned about your weight?	3	0.770
Do you deliberately eat foods that are slimming?	5	0.755
When you have put weight, do you eat less than you usually do?	1	0.736
Do you think that on the market there is also unhealthy food?	4	0.695
Cronbach’s alpha		0.928
Percentage of variances explained		60.69%

A confirmatory factor analysis was run on sample 2, using the structure obtained in Sample 1. The following results were obtained: the Maximum Likelihood Chi-Square = 318.56 and Degrees of Freedom = 35, which gave an × 2/df = 9.10. For non-centrality fit indices, the Steiger-Lind RMSEA was 0.118 [0.104–0.155]. Moreover, the Joreskog GFI equaled 0.917 and AGFI equaled 0.920.

### Test-retest reliability

The results of the test-retest reliability assessment demonstrated strong reproducibility of the Dutch restrained eating scale [Intraclass correlation (95% CI): ICC = 0.92 (0.83–0.96), *p* < 0.001].

### Bivariate analysis

A significantly higher mean restrained eating scale score was found in female, married participants compared to male and single ones (2.65 vs. 2.35 and 2.71 vs. 2.49 respectively) and in those with an intermediate income compared to participants without income (2.74 vs. 2.49). A significantly higher mean of restrained eating was seen in participants who practiced sport (2.68 vs. 2.36) compared to those who did not follow this habits. Also, a significantly higher mean restrained eating scale was found in participants that had pressure from TV/magazines to lose weight (3.09 vs. 2.43) and had a family history of eating disorders (2.85 vs. 2.47) compared to those who did not agree with these statements (Table 3).

**Table 3:** Bivariate analysis of the factors associated with the Dutch restrained eating scale

		Dutch restrained eating scale	*p*-value
		Mean ± SD	
Gender	Male	2.35 ± 0.92	< 0.001
	Female	2.65 ± 0.97	
Marital status	Single	2.49 ± 0.98	0.004
	Married	2.71 ± 0.91	
Monthly income	No income	2.49 ± 0.98	0.021
	< 1000$	2.50 ± 0.97	
	1000–2000 $	2.74 ± 0.91	
	> 2000 $	2.80 ± 0.83	
Education level	Primary	2.67 ± 1.02	0.926
	Complementary	2.51 ± 0.85	
	Secondary	2.56 ± 0.97	
	University	2.55 ± 0.97	
Alcohol use	Yes	2.55 ± 1.17	0.984
	No	2.54 ± 0.95	
Tobacco use	Yes	2.48 ± 0.99	0.173
	No	2.59 ± 0.95	
Caffeine	Yes	2.57 ± 0.97	0.053
	No	2.35 ± 0.90	
Family history of eating disorders	Yes	2.85 ± 0.90	< 0.001
	No	2.47 ± 0.97	
Pressure from TV, magazine to lose weight	Yes	3.09 ± 0.90	< 0.001
	No	2.43 ± 0.94	
Practicing sport activities	Yes	2.68 ± 0.95	< 0.001
	No	2.36 ± 0.96	
Receiving comments from the family concerning losing weight	Yes	2.64 ± 0.97	0.113
	No	2.52 ± 0.96	
Being insulted	Yes	2.65 ± 0.97	0.354
	No	2.54 ± 0.96	
Being physically abused	Yes	2.65 ± 0.91	0.405
	No	2.54 ± 0.97	
Being sexually abused	Yes	2.61 ± 0.84	0.741
	No	2.55 ± 0.97	

*SD* Standard deviation

In addition, a significant but low positive correlation was found between more restrained eating and increased age (r = 0.113), higher body dissatisfaction scores (r = 0.296), higher BMI (r = 0.063), higher ERQ (cognitive reappraisal facet) (r = 0.070), higher security attachment (r = 0.060), higher anxiety attachment (r = 0.154) and higher avoidance attachment (r = 0.082). However, a significant but low negative correlation was found between lower restrained eating scores and higher self-esteem scores (r = − 0.068) (Table 4).

**Table 4:** continuous variables associated with the Dutch restrained eating scale

	Correlation coefficient	*p*-value
Age	0.113	0.001
Body dissatisfaction score	0.296	< 0.001
Body Mass Index	0.063	0.080
ERQ cognitive reappraisal facet	0.070	0.065
ERQ expression suppression facet	0.050	0.189
State Adult attachment scale - Security	0.060	0.095
State Adult attachment scale - Anxiety	0.154	< 0.001
State Adult attachment scale - Avoidance	0.082	0.021
Self-esteem scale	−0.068	0.054
Depression	0.019	0.590
Anxiety	0.018	0.612
Stress	0.004	0.917
Emotional eating scale	−0.049	0.172
Physical activity index	0.057	0.187

*ERQ* Emotion Regulation Questionnaire

### Multivariable analysis

The results of a first linear regression, taking the Dutch restrained eating scale as the dependent variable and the sociodemographic as independent variables, showed that being a female (Beta = 0.461), higher BMI (Beta = 0.042) and intermediate and high monthly income (Beta = 0.273 and Beta = 0.344 respectively) were associated with higher restrained eating scores (Table 5, Model 1).

**Table 5:** Multivariable analysis

Model 1: Linear regression taking the Dutch restrained eating scale as the dependent variable and the sociodemographic variables as independent ones.					
	Unstandardized Beta	t	*p*-value	Confidence interval	
				Lower Bound	Upper Bound
Gender (females vs. males^a^)	0.461	6.257	<0.001	0.316	0.605
Body Mass Index	0.042	6.350	<0.001	0.029	0.055
High monthly income vs. no income^a^	0.344	2.425	0.016	0.066	0.623
Intermediate monthly income vs. no income^a^	0.273	2.825	0.016	0.066	0.623
Variables entered: Age, gender, BMI, marital status and monthly income. R^2^=0.086					
Model 2: Linear regression taking the Dutch restrained eating scale as the dependent variable and the pressure variables as independent variables.					
	Unstandardized Beta	t	*p*-value	Confidence interval	
				Lower Bound	Upper Bound
Body dissatisfaction score	0.038	6.176	<0.001	0.026	0.050
BMI	0.017	2.351	0.019	0.003	0.031
Practicing sport activities (yes vs. no^a^)	0.381	5.540	<0.001	0.246	0.516
Age	0.010	3.434	0.001	0.004	0.016
Gender (females vs. males^a^)	0.356	5.038	<0.001	0.217	0.494
Pressure from TV, magazine to lose your weight	0.484	5.478	<0.001	0.310	0.657
Intermediate monthly income vs. no income^a^	0.228	2.408	0.016	0.042	0.414
Variables entered = Age, gender, BMI, marital status, monthly income, caffeine, family history of eating disorders, Pressure from TV, magazine to lose your weight, Practicing sport activities and body dissatisfaction score. R^2^=0.211					
Model 3: Linear regression taking the Dutch restrained eating scale as dependent variable and all other scales as independent variables.					
	Unstandardized Beta	t	*p*-value	Confidence interval	
				Lower Bound	Upper Bound
Body dissatisfaction score	0.037	5.735	<0.001	0.024	0.049
Age	0.011	3.543	<0.001	0.005	0.017
Gender (females vs. males^a^)	0.316	4.240	<0.001	0.170	0.462
BMI	0.018	2.470	0.014	0.004	0.033
State Adult attachment scale-Anxiety	0.008	1.965	0.050	0.0003	0.015
Pressure from TV, magazine to lose your weight (yes vs. no^a^)	0.458	4.969	<0.001	0.277	0.639
Practicing sport activities (yes vs. no^a^)	0.414	5.724	<0.001	0.272	0.556
Emotion regulation cognitive reappraisal facet	0.014	2.876	0.004	0.004	0.023
Intermediate income vs. no income^a^	0.256	2.518	0.012	0.056	0.455
Variables entered in the model: Age, gender, BMI, marital status, monthly income, caffeine, family history of eating disorders, Pressure from TV, magazine to lose your weight, Practicing sport activities, self-esteem scale, ERQ cognitive reappraisal facet, SAAM-Anxiety, SAAM-avoidance, SAAM-security and Body dissatisfaction score. R^2^=0.238					

^a^Reference group; R^2^ = Nagelkerke value

A second linear regression, taking the Dutch restrained eating scale as the dependent variable and the pressure variables as independent variables showed that being a female (Beta = 0.356), ageing (Beta = 0.010), higher body dissatisfaction score (Beta = 0.03), higher BMI (Beta = 0.01), practicing sport activity (Beta = 0.38), intermediate monthly income (Beta = 0.22) and receiving pressure from TV/magazines to lose weight (Beta = 0.48) were associated with higher restrained eating scores (Table 5, Model 2).

A third linear regression, taking the Dutch restrained eating scale as the dependent variable and the emotion scales as independent variables, showed that being a female (Beta = 0.31), increased age (Beta = 0.01), higher BMI (Beta = 0.01), intermediate monthly income (Beta = 0.25), higher body dissatisfaction scores (Beta = 0.03), higher anxiety attachment (Beta = 0.008), ERQ cognitive reappraisal facet (Beta = 0.01), receiving pressure from TV/magazine to lose weight (Beta = 0.45) and practicing sport activities (Beta = 0.41) were associated with higher restrained eating scores (Table 5, Model 3).

## Discussion

To our knowledge, this is the first study in the country to determine factors associated with restrained eating. This project falls in a project about the validation of eating disorders scales and correlates associated with them in the Lebanese population [50–52]. The results revealed that being a female, increased age, intermediate monthly income, higher body dissatisfaction, higher anxiety attachment, higher emotional regulation cognitive reappraisal facet, exercising to lose weight, having pressure from TV/magazine to lose weight, dieting to lose weight and practicing sport activities were associated with more restrained eating.

### Validity of the Dutch restrained eating scale

In our study, the factor analysis showed that all items on restrained eating had high loadings on one factor as seen in the original Dutch version of the questionnaire, indicating a robust factorial validity [38]. Similarly, the same findings were also found by Wardle (1987) [53] and Lluch et al. (1996) [54] in their validation study into the English and French languages respectively. Moreover, the elevated Cronbach’s alpha coefficient found in our sample indicates that the scale has a high internal consistency. Similar results were found by Van Strien et al. in the original Dutch restrained eating scale, with alpha coefficients ranging in-between 0.80 and 0.95, as well as by Lluch et al. in the French version with results showing a mean alpha of 0.9. In addition, a confirmatory factor analysis was conducted in our study on another sample confirming the validity of the scale. Hence, the Arabic version of the Dutch Restrained Eating scale presents a good reliability, which is equivalent to the original Dutch version of the questionnaire and is a good tool to be used among the Lebanese population.

### Socio-demographic factors

In the present study, our findings showed that females were more likely to be involved in restrained eating than males, in agreement with previous findings that showed that women were more preoccupied with weight loss and diet [55], whereas men were more concerned with body building [56]. In addition, our findings consolidate the ones of another study [57] conducted among adult population showed that restrained eating is much more common in women. Men, however, control their weight with exercising and implement diets only for health reasons. Women are more often affected by problems with their eating behavior, such as craving for special foods, than men.

As for exercising to lose weight, it was linked to increased restraint eating according to our results, in agreement with a previous study conducted in 2008 that showed that exercise was able to favorably modify the short-term appetite control [58]. The association between restrained eating and physical activity in determining energy intake after exercise remains unclear and may be related to disinhibition (loss of restraint) levels [59]. Acute exercise in restrained eaters is more effective in creating a negative energy balance as opposed to unrestrained eaters who actually increase their energy intake after physical activity whereas restrained eaters tend to decrease their energy intake after exercise [60]. More studies are recommended to solve the mystery of this dilemma.

Moreover, our results show a significant relationship between higher age and increased restrained eating patterns. This finding is in agreement with previous research where middle-aged women presented evidence of disordered eating [61]. The pathology behind it was linked to similar elements associated to eating disorders in younger women, with a focus on body dissatisfaction and sociocultural pressures [62].

Furthermore, in the current study, a significant positive association between BMI and restrained eating was seen, concurring previous research findings [14, 63] and confirming the results of a study conducted on Lebanese and Cypriot female students in 2012 [16]. In addition, a longitudinal study conducted by Snoek H.M. also found a positive correlation between BMI and restrained eating and revealed that higher BMI predicts more restrained eating [15].

### Body dissatisfaction and pressures

Restrained diet was reinforced by a body dissatisfaction according to cognitive behavioral models [64]. Our findings show a significant positive relationship between body dissatisfaction and restrained eating, in line with an increasing number of studies [65, 66]. Persons who worry about their weight and those who have high levels of fat in their body would have a greater body dissatisfaction, and consequently turn out to the restrained eating aiming at controlling their weight [67].

Furthermore, our results showed a positive correlation between restrained eating and social media pressure. In fact, several studies, including one conducted in Lebanon [65], have demonstrated the widespread of body image concerns due to the interrelated effects of economic, cultural, and media globalization [68, 69]. The latter have played a major role in promoting unrealistic body ideal among males and females. This concerned group are more prone to experience more body dissatisfaction and engage in multiple behaviors including but not limited to eating disorders, cosmetic surgeries in order to reach their ideal body image engraved in their heads [70].

### Emotion-related factors

Our results showed a significant association between the cognitive reappraisal facets and restrained eating, suggesting that restrained eaters have a higher tendency to use it as an emotion regulation strategy. They further consolidate previous findings [71, 72] that showed the well-known importance of emotional dysregulation on body image structure. In fact, greater emotional regulation difficulties and body dissatisfaction were significant predictors of disordered eating [73]. Emotional stress increases food intake as suggested by the restraint theory [74]. Restrained eaters see themselves forbidden to continue their diet when they have negative emotions since they find themselves busy in more “urgent concerns” and their cognitive function limited by distraction (whether by emotional matters or not) [75].

Moreover, reappraisal can be regarded as an adaptive emotion regulation strategy [76]. There are two interacting self-regulatory systems, a “cool” cognitive system and a “hot” emotional system according to the hot–cool system framework [77]. It looks like “hot” individuals focus on prompt satisfaction and are not able to control their eating habits compared to the “cool” ones.

Regarding adult attachment, an anxiety adult attachment style was correlated to a higher restrained eating, in accordance with a study conducted by Evens and Wetheim (1998) [78] who found that women with eating disorders, who thrive for thinness, reported an insecure attachment style. Similarly, other findings have shown the link between anxious attachments, avoidant attachment and disordered eating [79]. Moreover, a study by Broberg, Hjamlmers, and Nevonen [80] revealed that the majority of women who had a secure attachment style, never had an eating disorder. This secure attachment style was also linked to a greater body satisfaction [81]. Adult attachment preoccupation is characterized by “high anxiety, ambivalence, dependence and repressed anger and fear of losing the object” [82]. Under stressful situations, those individuals behave in a hyper-active way, which results in resilient negative thoughts, moods, emotion dysregulation, and maladaptive coping mechanisms [83]. Emotional eating can be viewed as a maladaptive attempt to reduce negative emotions [84].

Furthermore, previous findings have shown low to moderate positive associations between restraint and emotional eating [85]. A study conducted on Lebanese and Cypriot students in 2012 demonstrated a positive relationship between these 2 patterns [16] which contradicts the findings of our study where no significant association was found. On the other hand, the above mentioned studies have suggested that emotional eating may characterize only a subgroup of dieters which could explain our results. More studies are necessary to support these findings.

### Implications

Several factors might be associated with restrained eating according to our findings, which emphasize on the importance of preventing these factors rather than restrained eating itself. The finding that restrained eating might be driven by emotional regulation, attachment styles and other factors are actually promising results; although avoiding negative emotions and stressful conditions is almost impossible, learning how to regulate one’s emotions and deal with stressors may be essential in removing a crucial eating disorder’s instigator.

## Limitations

The current study was a cross-sectional one and therefore, could not assess causality of relationships. Some of the scales used in this study have not been validated in Arabic yet. Participants were diagnosed using a score tool and not through a clinical diagnostic interview, therefore, we could not confirm the accuracy of responses. Further assessment by a psychiatrist or a psychologist is needed to confirm the results obtained. Females were more represented numerically than males; future studies enrolling an equal number of males and females is needed to evaluate the association between gender and restrained eating. The results obtained in this study might not be representative of the whole population since a high percentage of the participants (73.2%) had a high level of education. Another limitation concerns the lower reliability in men than women [85] and that both genders differ significantly on the body dissatisfaction and drive for thinness scales of this inventory [86]. These differences are not surprising as the items of these scales were tailored to assess discontentment with weight, overall shape and body parts from the waist-down, and fear of gaining weight and desire to be thinner [87]. In order to be appropriate for men, body image assessment tools must contain several items that address attitudes toward muscularity as well as items exploring attitudes toward upper body parts [88]. Furthermore, despite the documented qualitative gender differences in the perceptions of overall ideal body shape and specific body areas of concern [89, 90], the majority of available instruments have been specifically developed for women and are not valid for men [88, 89].

## Conclusion

In conclusion, our study revealed that factors associated with restrained eating in the Lebanese community include body dissatisfaction, cognitive reappraisal, female gender, eating attitudes, social media pressure and adult attachment. Since dietary restriction has been linked to many clinically diagnosed eating disorders, the current findings might have significant impact on the development of prevention strategies targeted towards a misperception regarding body image; improving knowledge about factors that might influence this body image and critical thinking regarding media images is warranted, with the ultimate goal of promoting healthier choices in the Lebanese population.

## Data Availability

The authors do not have the right to share any data information as per their institutions policies.

## Abbreviations

AGFI: Adjusted Goodness of Fit Index
AN: Anorexia nervosa
ANOVA: Analysis of variance
BED: Binge eating disorder
BES: Binge Eating Scale
BMI: Body Mass Index
BN: Bulimia nervosa
DSM-4: Fourth Edition of the Diagnostic and Statistical Manual of Mental Disorders
DSM-5: Diagnostic and Statistical Manual of Mental Disorders, Fifth Edition
ED: Eating disorder
EDI-2: Body dissatisfaction subscale of the Eating Disorder Inventory-second version
EDNOS: Eating disorder not otherwise specified
EES: Emotional eating scale
ERQ: Emotion Regulation Questionnaire
GFI: Goodness of Fit Index
HAM-A: Hamilton Anxiety Rating Scale
HAM-D: Hamilton Scale for Depression
KMO: Kaiser–Meyer–Olkin
PSS: Perceived Stress Scale
RMSEA: Root Mean Square Error of Approximation
SAAM: State adult attachment measure
